# The Great Mimickers: Navigating Diagnostic Pitfalls in Modern Rheumatology

**DOI:** 10.7759/cureus.113645

**Published:** 2026-07-30

**Authors:** Yarden Assabag

**Affiliations:** 1 Rheumatology, Internal Medicine, Clalit Health Services, Beersheba, ISR

**Keywords:** clinical reasoning, diagnostic mimickers, differential diagnosis, systemic autoimmunity, systemic lupus erythematosus

## Abstract

Background

Rheumatological mimics frequently cause diagnostic errors and unnecessary immunosuppression due to overreliance on positive serology or non-specific imaging. This review evaluates eight illustrative cases where initial objective findings heavily conflicted with the final, etiology-driven diagnosis.

Methods

A retrospective chart review of eight patients from a tertiary rheumatology practice was performed. Cases were selected based on significant incongruence between initial laboratory or high-sensitivity imaging profiles and the final clinical etiology. A literature search across PubMed and Google Scholar up to June 2026 contextualized these pitfalls.

Results

The cohort highlighted critical diagnostic traps: crystal arthropathy misdiagnosed as systemic vasculitis, lupus phenocopies, and structural/functional disorders (e.g., temporomandibular joint dysfunction) misattributed to fibromyalgia. Comprehensive clinical re-evaluation led to the cessation of inappropriate immunomodulatory treatments and the initiation of targeted, etiology-specific therapies. Comprehensive re-evaluation led to the cessation of inappropriate immunomodulatory treatments in all eight patients, with six receiving targeted etiology-specific therapies and two benefiting from lifestyle or occupational modifications.

Conclusions

Isolated laboratory titers and imaging findings must never supersede strict clinical correlation. Recognizing these common mimics is essential to halt diagnostic momentum and prevent treatment-related morbidity.

## Introduction

The diagnosis of systemic autoimmune diseases has been fundamentally revolutionized by high-sensitivity imaging modalities and expanded autoantibody profiling. In an era increasingly dominated by defensive medicine and standardized diagnostic criteria protocols, there is an expanding clinical tendency to treat an isolated laboratory titer or radiological report rather than the comprehensive patient [1].

Rheumatological mimics-conditions that accurately simulate complex autoimmune pathology through alternative metabolic, occupational, or reactive pathways-are frequently overlooked in busy clinical settings [2]. This diagnostic oversight is especially prevalent when clinician evaluation becomes overly reliant on strict diagnostic criteria [3]. When an objective biomarker is misinterpreted out of its clinical context, it frequently triggers an unintended cascade of diagnostic momentum. These diagnostic "anchors" often lead to inappropriate, long-term immunosuppressive therapy, severe treatment-related toxicity, profound psychological distress as patients grapple with the uncertainty of a chronic autoimmune label that may later be overturned, and critical delays in identifying the true underlying etiology [4,5].

Ultimately, this technological leap has introduced a distinct clinical challenge: the "diagnostic pitfall" [6]. In this clinical review, I will present a series of eight complex diagnostic encounters where initial serological or high-sensitivity imaging profiles heavily conflicted with the final clinical etiology. From parathyroid-driven crystal arthropathies mimicking refractory gout to systemic chronic inflammation inducing serological false positives, these cases highlight the necessity of maintaining a high index of clinical suspicion. All scenarios presented herein are drawn from real-world tertiary and community rheumatology practices, serving to underscore the primacy of meticulous bedside reasoning over uncritical biomarker reliance, keeping in mind both false-positive and false-negative pitfalls.

## Materials and methods

This retrospective case-based review evaluates eight illustrative diagnostic encounters from a tertiary rheumatology practice, selected due to significant incongruence between initial serology or high-sensitivity imaging and the final clinical diagnosis. Highlighted pitfalls include crystal arthropathies, lupus phenocopies, and misdiagnosed fibromyalgia. To ensure patient confidentiality, all clinical data were fully anonymized in accordance with the Declaration of Helsinki. Due to the retrospective and fully anonymized nature of this chart review, it was exempt from institutional ethical review, and written informed consent was waived.

A comprehensive literature search was conducted across PubMed and Google Scholar databases up to June 2026 using MeSH terms and keywords including "rheumatologic mimics," "diagnostic pitfalls," and "overdiagnosis in autoimmunity." The search was limited to peer-reviewed English publications, focusing on consensus guidelines and mechanistic reviews. The clinical characteristics and key diagnostic findings of the cohort are summarized in Table 1 and Table 2.

**Table 1:** Comprehensive summary of case cohort, laboratory findings, and clinical pearls CPPD - calcium pyrophosphate deposition; PTH - parathyroid hormone; SLE - systemic lupus erythematosus; CPK - creatine phosphokinase; anti-HMGCR - anti-3-hydroxy-3-methylglutaryl-CoA reductase; HS - hidradenitis suppurativa; PMR - polymyalgia rheumatica

Case / condition	Key symptoms & clinical presentation	Key laboratory findings	Reference range & units	Final diagnosis & treatment	Clinical pearl / diagnostic mirage
Case one: CPPD disease	Episodic, oligoarticular inflammatory arthritis in knees, ankles, and wrists (4-5 times/year).	Serum uric acid: 7.4 mg/dL	3.5-7.0 mg/dL	Primary hyperparathyroidism & CPPD. Treatment: surgical resection of parathyroid adenoma; cessation of unnecessary urate-lowering therapy.	Oligoarthritis in large joints - especially when refractory to standard gout treatment - must trigger a metabolic workup (PTH, calcium, magnesium) before committing to lifelong urate-lowering therapy.
		Total serum calcium: 11.2 mg/dL	8.5-10.5 mg/dL		
		PTH: 118 pg/mL	15-65 pg/mL		
		Serum magnesium: 1.3 mg/dL	1.7-2.2 mg/dL		
Case two: silica SLE phenocopy	Inflammatory polyarthritis and profound fatigue; history of high-intensity silica dust exposure.	Antinuclear antibody: 1:1280	Negative (<1:160), complement levels (C3, C4): normal; Urinalysis: normal, negative for proteinuria	Silica-induced systemic autoimmunity. Treatment: strict occupational exposure mitigation and dust cessation; avoided long-term aggressive immunosuppression.	Clinicians should exercise caution when assigning a diagnosis of idiopathic SLE in the presence of high-risk occupational exposures, even when "pathognomonic" antibodies likeanti-Smith are strongly positive.
		Anti-Smith (anti-Sm): 85 U/mL	<20 U/mL		
		Anti-double-stranded DNA: 160 IU/mL	<30 IU/mL		
Case three: anti-HMGCR mirage	Progressive lower limb pain, bilateral thigh muscle edema on MRI; background of severe grade IV HS.	CPK: 115 U/L	39-308 U/L	Bilateral avascular necrosis (AVN) & serological false-positive. Treatment: Avoided myositis immunosuppression; targeted management for AVN and underlying HS.	In patients with severe systemic inflammation, a positive autoantibody with an entirely normal CPK level should immediately prompt a careful search for alternative structural mimics or immunoassay interference.
		Erythrocyte sedimentation rate: 95 mm/hr	<20 mm/hr		
		Anti-HMGCR antibodies: >100 U/mL	<20 U/mL		
Case four: structural myopathy	Three-year progressive lower limb weakness, diffuse muscle 'edema' and fatty replacement on MRI.	CPK: normal	39-308 U/L	Structural myopathy (limb-girdle muscular dystrophy). Treatment: discontinuation of ineffective three-year steroid/immusen regimen; physical therapy.	The persistence of a stable "edema" signal on MRI over years in a patient with a normal CPK is a major red flag. If a patient fails to respond to steroids, the clinician must pivot immediately to consider structural or genetic myopathies.
		Inflammatory markers: Normal	Varies by assay		
Case five: toxic rhabdomyolysis	Acute, severe muscle pain, symmetrical proximal weakness, and dark urine in a competitive bodybuilder.	CPK: >25,000 U/L	39-308 U/L	Anabolic-steroid induced mechanical rhabdomyolysis. Treatment: Aggressive intravenous hydration, metabolic monitoring, and strict cessation of anabolic-androgenic steroids (AAS).	Commencing aggressive immunosuppression based solely on extreme CPK elevations without an exhaustive lifestyle audit can be a dangerous diagnostic error; a thorough substance-use history must precede invasive testing.
		Autoantibody panel: Negative	Negative		
Case six: vibration-induced	Two-year history of episodic cold-induced digital blanching, cyanosis, and pain (Raynaud's).	Antinuclear antibody: 1:160	Negative (<1:160)	Hand-arm vibration syndrome (HAVS) mimicking scleroderma. Treatment: Occupational mitigation (vibration cessation); avoided futile vasodilatory or immunomodulatory therapy.	Raynaud's phenomenon coupled with a low-titer antinuclear antibody can act as a diagnostic decoy; the definitive intervention for occupational microvascular damage is exposure mitigation, not futile immunomodulation.
		Scleroderma-specific antibodies: Negative	Negative		
Case seven: paraneoplastic PMR	Proximal girdle pain, severe morning stiffness, initial rapid response to 15mg prednisone, later relapse.	Erythrocyte sedimentation rate: 75 mm/hr (initial)	<20 mm/hr	Malignancy-associated paraneoplastic polymyalgic syndrome. Treatment: referral to gynecologic oncology for advanced ovarian carcinoma management; corticosteroid taper.	The rapid therapeutic response to low-dose steroids is a cognitive trap (the steroid-confirmation fallacy) and is not a specific test for PMR; a lack of sustained response during tapering must prompt malignancy screening.
		Abdominal/Pelvic CT Findings: Positive	N/A		
Case eight: Nutritional Pain	Five-year history of widespread musculoskeletal pain, chronic fatigue, non-restorative sleep, and brain fog.	Vitamin D (25-OH): 8 ng/mL	>30 ng/mL	Profound multiple nutritional deficiencies mimicking fibromyalgia. Treatment: aggressive targeted nutritional repletion (vitamin D, iron, B12), leading to a 90% pain reduction.	Fibromyalgia should not be used as a diagnostic wastebasket before conducting a fundamental metabolic and nutritional audit; severe deficiencies in vitamin D, iron, and B12 can fully simulate centralized pain.
		Serum ferritin: 6 ng/mL	10-120 ng/mL		
		Vitamin B12: 110 pg/mL	211-911 pg/mL		

**Table 2:** Summary of rheumatological phenocopies and key clinical pearls AAS - anabolic-androgenic steroids; ANA - antinuclear antibodies; anti-HMGCR - anti-3-hydroxy-3-methylglutaryl-CoA reductase; anti-Sm - anti-Smith; AVN - avascular necrosis; CPK - creatine phosphokinase; CPPD - calcium pyrophosphate deposition; ESR - erythrocyte sedimentation rate; FMS - fibromyalgia syndrome; HAVS - hand-arm vibration syndrome; HS - hidradenitis suppurativa; ILD - interstitial lung disease; IMNM - immune-mediated necrotizing myopathy; LGMD - limb-girdle muscular dystrophy; MRI - magnetic resonance imaging; MTP - metatarsophalangeal; PMR - polymyalgia rheumatica; SLE - systemic lupus erythematosus

Clinical Pearl	Diagnostic mirage / mimic
Oligoarthritis in large joints—especially when refractory to standard gout treatment—must trigger a metabolic workup (PTH, Calcium, Magnesium) before committing to lifelong urate-lowering therapy	Case one: CPPD disease masquerading as refractory gout
Clinicians should exercise caution when assigning a diagnosis of idiopathic SLE in the presence of high-risk occupational exposures, even when "pathognomonic" antibodies like Anti-Smith are strongly positive.	Case two: silica-induced SLE phenocopy
In patients with severe systemic inflammation, a positive autoantibody with an entirely normal CPK level should immediately prompt a careful search for alternative structural mimics or immunoassay interference.	Case three: anti-HMGCR false positive & AVN
The persistence of a stable "edema" signal on MRI over years in a patient with a normal CPK is a major red flag. If a patient fails to respond to steroids, the clinician must pivot immediately to consider structural or genetic myopathies. In such cases, early referral for genetic counseling and testing can prevent years of ineffective immunosuppression and guide appropriate rehabilitation.	Case four: structural myopathy (LGMD) mimicking Myositis
Commencing aggressive immunosuppression based solely on extreme CPK elevations without an exhaustive lifestyle audit can be a dangerous diagnostic error; a thorough substance-use history must precede invasive testing.	Case five: anabolic-induced rhabdomyolysis
Raynaud's phenomenon coupled with a low-titer ANA can act as a diagnostic decoy; the definitive intervention for occupational microvascular damage is exposure mitigation, not futile immunomodulation.	Case six: hand-arm vibration syndrome (HAVS) mimicking scleroderma
The rapid therapeutic response to low-dose steroids is a cognitive trap (the steroid-confirmation fallacy) and is not a specific test for PMR; a lack of sustained response during tapering must prompt malignancy screening.	Case seven: malignancy-associated paraneoplastic syndrome
Fibromyalgia should not be used as a diagnostic wastebasket before conducting a fundamental metabolic and nutritional audit; severe deficiencies in Vitamin D, Iron, and B12 can fully simulate centralized pain.	Case eight: nutritional deficiencies mimicking fibromyalgia

## Results

The clinical profiles, initial diagnostic pitfalls, and final etiology-driven diagnoses of the eight illustrative cases are summarized in Table 1. To preserve clinical clarity and maintain the narrative integrity of each diagnostic encounter, each case is presented chronologically below, incorporating its specific clinical presentation, diagnostic challenge, and subsequent pathophysiological synthesis.

Case one: when "gout" is an endocrine signal - primary hyperparathyroidism

Clinical Presentation

A 54-year-old male presented with episodic, oligoarticular inflammatory arthritis, occurring four to five times per year. The flares primarily involved the knees, ankles, and wrists. Based on a history of borderline hyperuricemia (serum uric acid: 7.4 mg/dL; normal range: 3.5-7.0 mg/dL), the patient had been managed for years as "refractory gout," yet the frequency of flares remained unchanged despite urate-lowering therapy. Targeted metabolic screening later uncovered significant hypercalcemia (total serum calcium: 11.2 mg/dL; normal range: 8.5-10.5 mg/dL), elevated parathyroid hormone (PTH: 118 pg/mL; normal range: 15-65 pg/mL), and concurrent hypomagnesemia (serum magnesium: 1.3 mg/dL; normal range: 1.7-2.2 mg/dL).

The Diagnostic Challenge

The distribution of the arthritis - favoring large joints rather than the first metatarsophalangeal (MTP) joint - raised suspicion that the gout diagnosis was a "diagnostic anchor." In the community clinic, hyperuricemia is frequently used as a surrogate for gout, leading to the potential oversight of other crystal arthropathies. A critical review of the radiographs was performed.

Pathophysiological Synthesis & Discussion

The review identified subtle but definitive chondrocalcinosis (calcification of the hyaline cartilage) in the knees and wrist joints. This finding shifted the diagnosis from gout to calcium pyrophosphate deposition (CPPD) disease. Recognizing that CPPD is a classic phenocopy of gout that often mirrors underlying metabolic derangement, targeted screening uncovered significant hypercalcemia and hypomagnesemia.

This case underscores the role of CPPD as a "metabolic red flag." While gout is a disorder of urate metabolism, CPPD is frequently triggered by hyperparathyroidism, hypomagnesemia, or hemochromatosis [1]. In this patient, a parathyroid adenoma led to primary hyperparathyroidism, which altered calcium-pyrophosphate homeostasis and promoted crystal precipitation in the joint space. The diagnostic pitfall was the "borderline hyperuricemia," which acted as a decoy. In modern practice, oligoarthritis in large joints-especially when refractory to standard gout treatment-must trigger a metabolic workup incorporating parathyroid hormone (PTH), calcium, and magnesium before committing to lifelong urate-lowering therapy [2]. Furthermore, serum phosphorus and 24-hour urinary calcium in the workup can help differentiate primary hyperparathyroidism from benign familial hypocalciuric hypercalcemia, avoiding unnecessary surgery.

Case two: the silica-induced SLE phenocopy - beyond the anti-Smith paradigm

Clinical Presentation

A 40-year-old male, owner of a stone-processing factory with decades of high-intensity silica dust exposure, presented with clinical features highly suggestive of systemic lupus erythematosus (SLE), including inflammatory polyarthritis and profound fatigue. The serological profile was remarkable, showing a high-titer antinuclear antibody (ANA 1:1280, speckled pattern) and, crucially, strongly positive anti-Smith (anti-Sm) antibodies (85 U/mL; normal: <20 U/mL) and anti-double-stranded DNA (anti-dsDNA) antibodies (160 IU/mL; normal: <30 IU/mL).

The Diagnostic Challenge

In current rheumatological practice, anti-Smith antibodies are considered nearly 100% specific for idiopathic SLE and are a cornerstone of the European Alliance of Associations for Rheumatology/American College of Rheumatology (EULAR/ACR) classification criteria. However, this case presents a profound "diagnostic mirage". While the patient technically met the criteria for SLE, his extensive occupational history suggested an alternative driver: silica-induced systemic autoimmunity.

Pathophysiological Synthesis & Discussion

Silica is a potent crystalline adjuvant that triggers the NLRP3 inflammasome within alveolar macrophages. This leads to chronic inflammatory cell death (pyroptosis) and the subsequent "shedding" of intracellular antigens. In genetically susceptible individuals, this sustained antigenic challenge can induce a serological profile that either acts as a diagnostic phenocopy or triggers true systemic lupus erythematosus [3, 4]. A detailed occupational history, using a standardized questionnaire, should be considered a routine part of the initial evaluation for any patient presenting with suspected SLE [3, 4].

The case of silica-induced autoimmunity represents a profound phenocopy of systemic lupus erythematosus. While anti-Smith antibodies are traditionally viewed as pathognomonic for idiopathic SLE, the adjuvant effect of crystalline silica can bypass normal immune tolerance [4]. As demonstrated in previous cohort studies of exposed workers, the molecular mimicry and chronic antigenic shedding triggered by silica particles can induce a serological profile that is indistinguishable from idiopathic disease [3]. Clinicians should exercise caution when assigning a diagnosis of idiopathic SLE in the presence of high-risk occupational exposures, even when "pathognomonic" antibodies like anti-Sm are present.

Case three: the HMGCR mirage - pseudo-myositis in chronic inflammatory states

Clinical Presentation

A 36-year-old patient with grade IV hidradenitis suppurativa (HS), refractory to multiple biologic therapies, presented with progressive lower limb pain. Initial evaluation was alarming: magnetic resonance imaging (MRI) of the thighs demonstrated extensive bilateral muscle edema, and serological screening returned a strong positive result for anti-3-hydroxy-3-methylglutaryl-CoA reductase (anti-HMGCR) antibodies (>100 U/mL; normal: <20 U/mL). However, the patient's creatine phosphokinase (CPK) levels were consistently normal (115 U/L; normal range: 39-308 U/L), and inflammatory markers revealed extreme hypergammaglobulinemia with an erythrocyte sedimentation rate (ESR) of 95 mm/hr.

The Diagnostic Challenge

The combination of muscle edema on MRI and a positive Anti-HMGCR antibody typically mandates a diagnosis of immune-mediated necrotizing myopathy (IMNM). However, the clinical picture was discordant: the patient's creatine phosphokinase (CPK) levels were consistently normal, and physical examination revealed preserved proximal muscle strength-findings fundamentally inconsistent with active necrotizing myositis.

Pathophysiological Synthesis & Discussion

A dedicated musculoskeletal review identified bilateral avascular necrosis (AVN) of the femoral heads, likely a consequence of long-term corticosteroid use and chronic systemic inflammatory stress. The muscle edema on MRI was re-interpreted as reactive "neighboring" edema from the overlying cutaneous sinus tracts and the underlying joint pathology, rather than primary autoimmune myopathy. Anti-HMGCR myopathy is a necrotizing process characterized by profound muscle fiber destruction, typically resulting in creatine phosphokinase (CPK) elevations 10 to 50 times the upper limit of normal [5]. In this patient, the persistently normal CPK was the key clinical "red flag" against the serological result. When diagnostic uncertainty persists, a muscle biopsy may provide definitive evidence to rule out myositis and prevent unnecessary immunosuppression.

The positive Anti-HMGCR antibody may represent a false-positive finding secondary to assay interference rather than true active myopathy. It is well-documented that extreme hypergammaglobulinemia (reflected here by an erythrocyte sedimentation rate (ESR) >90 mm/hr) can interfere with solid-phase immunoassays (enzyme-linked immunosorbent assay (ELISA)/immunoblot) through non-specific protein binding, leading to false-positive results [6]. Furthermore, muscle edema on MRI is a non-specific marker; in the context of severe HS, it reflected "neighboring edema" from adjacent skin infection rather than primary inflammation. A positive autoantibody with a normal CPK should prompt a search for alternative mimics like AVN or reactive edema before escalating immunosuppression.

Case four: the three-year "myositis" mirage - structural myopathy vs. active inflammation

Clinical Presentation

A 35-year-old female patient was managed for three years under the presumptive diagnosis of inflammatory myositis. The diagnosis was primarily based on MRI findings of diffuse muscle 'edema' and progressive atrophy. Over these three years, she underwent multiple lines of immunosuppressive therapy with no clinical improvement.

The Diagnostic Challenge

A critical clinical audit revealed a profound "clinical silence" that contradicted the imaging. Despite the long duration of symptoms, the patient had no skin involvement, no interstitial lung disease (ILD), and no constitutional symptoms. Most importantly, her muscle enzymes (CPK) and inflammatory markers were consistently normal, and her gross motor strength remained remarkably preserved despite the "alarming" MRI.

Pathophysiological Synthesis & Discussion

The "bright signal" on MRI was misidentified as active inflammatory edema. In T2-weighted sequences, both water (edema) and lipids (fatty infiltration) can appear hyperintense. While fat-suppression techniques like short tau inversion recovery (STIR) are designed to isolate the water signal, chronic fatty replacement-a hallmark of structural myopathies like limb-girdle muscular dystrophy (LGMD)-can create a "pseudoinflammatory" appearance. This occurs when the altered muscle architecture prevents complete fat suppression, mimicking the high water content of active myositis [7, 8].

This case highlights the "MRI-First" diagnostic trap. The persistence of a stable "edema" signal over three years in a patient with a "quiet" laboratory profile (normal CPK) is a major red flag for a non-inflammatory etiology. Relying on imaging while ignoring the lack of clinical and biochemical markers leads to "diagnostic anchoring," resulting in years of ineffective and potentially toxic immunosuppressive therapy. Chronic, stable muscle symptoms coupled with a normal CPK should prompt a careful review of a 'noisy' MRI. If a patient with presumed myositis fails to respond to steroids and lacks systemic features, clinicians should keep structural or genetic myopathies high on the differential diagnosis, pending confirmation by further evaluation.

Case five: the anabolic-induced myalgia - rhabdomyolysis masquerading as polymyositis

Clinical Presentation

A 28-year-old male competitive bodybuilder presented with acute, severe muscle pain, symmetrical proximal muscle weakness, and dark urine. Laboratory evaluation revealed an extraordinarily elevated CPK level exceeding 25,000 U/L, raising immediate concern for acute polymyositis or necrotizing autoimmune myopathy.

The Diagnostic Challenge

An elevated CPK in a patient presenting with muscle pain and weakness typically triggers an immediate autoimmune myopathy protocol. However, a detailed lifestyle audit revealed a crucial confounding factor: the patient admitted to the heavy use of anabolic-androgenic steroids (AAS) combined with intense, eccentric resistance training.

Pathophysiological Synthesis & Discussion

This presentation represents a severe phenocopy of inflammatory myositis caused by toxic-mechanical rhabdomyolysis. Anabolic steroids accelerate muscle hypertrophy but can compromise myofibrillar structural integrity under extreme mechanical stress, inducing severe rhabdomyolysis [9]. Commencing aggressive immunosuppression or high-dose corticosteroids based solely on the CPK level would be a dangerous diagnostic error, as steroids can worsen muscle wasting and exacerbate metabolic derangements in an already compromised muscular system. In young, muscular individuals, a thorough diagnostic audit-incorporating medication and supplement history, substance use, infection, heat exposure, trauma, metabolic disorders, and a family history-must precede any autoantibody panel or muscle biopsy. Urine myoglobin testing, alongside early assessment of renal function and electrolytes, provides rapid confirmation of rhabdomyolysis and guides the need for aggressive hydration and renal monitoring.

Case six: the vibration-induced mirage - secondary Raynaud's phenomenon

Clinical Presentation

A 45-year-old male construction worker presented with a two-year history of episodic, cold-induced digital blanching followed by cyanosis and pain in both hands, classic for Raynaud's phenomenon. An initial laboratory screening returned a low-titer positive ANA (1:160), leading to a presumptive diagnosis of early systemic sclerosis (scleroderma).

The Diagnostic Challenge

The presence of Raynaud's phenomenon coupled with a positive ANA is a strong clinical signal for an evolving systemic autoimmune disease. However, a dedicated occupational audit revealed that the patient had spent over 15 years operating high-vibration industrial machinery, specifically jackhammers and heavy drills.

Pathophysiological Synthesis & Discussion

The patient's symptoms were driven by hand-arm vibration syndrome (HAVS)-an occupational disorder where prolonged mechanical vibration induces irreversible endothelial damage, microvascular hyperreactivity, and peripheral neuropathy [10, 11]. This structural vasospastic disease perfectly mimics the Raynaud's phenomenon seen in connective tissue diseases. To differentiate HAVS from true systemic sclerosis, a normal nailfold capillaroscopy and negative systemic sclerosis-related autoantibodies confirmed that the low-titer ANA was a benign serological finding, which is prevalent in up to 15% of the healthy population and often acts as a diagnostic decoy.

Labeling this patient with scleroderma would lead to unnecessary anxiety and futile vasodilatory or immunosuppressive therapies. The definitive intervention for HAVS is occupational mitigation and vibration cessation, not immunomodulation.

Case seven: the malignancy-induced "PMR" - the steroid-confirmation fallacy

Clinical Presentation

A 68-year-old female presented with a three-month history of profound bilateral shoulder and pelvic girdle pain, severe morning stiffness, and an elevated erythrocyte sedimentation rate (ESR) of 75 mm/hr. She was diagnosed with polymyalgia rheumatica (PMR) and started on low-dose prednisone (15 mg/day), which resulted in a dramatic, near-complete resolution of her pain within 48 hours.

The Diagnostic Challenge

The rapid therapeutic response to low-dose steroids is often viewed by clinicians as a diagnostic confirmation of PMR-a cognitive trap known as the "steroid-confirmation fallacy." However, four months into tapering her steroids, her symptoms returned aggressively, accompanied by unexpected weight loss and a further spike in inflammatory markers.

A comprehensive diagnostic re-evaluation-including age- and sex-appropriate screening guided by clinical presentation-revealed an underlying advanced ovarian carcinoma, confirming a paraneoplastic polymyalgic syndrome as a phenocopy of idiopathic PMR [12, 13]. Although the patient fulfilled standard classification criteria and alternative mimics were systematically excluded, corticosteroids acted as non-specific anti-inflammatory agents that masked systemic symptoms and created a false sense of diagnostic security. Clinicians must remember that a positive steroid response is non-specific; a lack of sustained response during tapering or the emergence of atypical constitutional symptoms should immediately trigger malignancy screening. Crucially, a marked clinical improvement in her musculoskeletal symptoms was observed following cancer-directed therapy.

Case eight: the "fibromyalgia" wastebasket - the nutritional deficiency trap

Clinical Presentation

A 42-year-old female presented with a five-year history of diffuse, widespread musculoskeletal pain, chronic fatigue, non-restorative sleep, and cognitive fog. She had been evaluated by multiple clinics and carried a firmly established diagnosis of "refractory fibromyalgia," managed with gabapentinoids and antidepressants with minimal benefit.

The Diagnostic Challenge

Fibromyalgia is frequently used as a diagnostic "wastebasket" for chronic pain syndromes when standard autoimmune markers are negative. The primary pitfall in this long-standing diagnosis was the absence of a fundamental metabolic and nutritional audit before concluding the pain was idiopathic.

Pathophysiological Synthesis & Discussion

A targeted metabolic screening revealed profound, concurrent nutritional deficiencies: her vitamin D level was extremely low (8 ng/mL), her ferritin was 6 ng/mL (severe iron deficiency), and her Vitamin B12 was 110 pg/L. Severe hypovitaminosis D induces chronic osteomalacia and generalized bone pain; iron deficiency compromises muscular mitochondrial function and causes profound fatigue; and vitamin B12 deficiency drives metabolic neuropathy and cognitive dysfunction. While these severe nutritional deficiencies can sometimes coexist with primary fibromyalgia or serve as major compounding factors rather than a standalone exclusive cause, together they created a distinct clinical phenocopy that perfectly simulated fibromyalgia syndrome (FMS). Following six months of aggressive nutritional repletion, objective documentation using validated scales (such as the Fibromyalgia Impact Questionnaire, FIQ) demonstrated a 90% reduction in pain scores and a complete resolution of her fatigue. This highlights the necessity of a thorough nutritional audit before cementing a diagnosis of fibromyalgia [14, 15].

## Discussion

The "diagnostic mirage" and diagnostic momentum

As demonstrated across the eight illustrative encounters presented in this review, high-sensitivity imaging and expanded serologic testing can inadvertently generate a "diagnostic mirage". When objective laboratory biomarkers or "noisy" imaging reports are prioritized over strict clinical congruence, it leads to diagnostic anchoring and unnecessary, potentially toxic immunosuppressive therapy.

Our cohort’s findings align with a growing body of literature highlighting diagnostic errors in autoimmunity. Epidemiological data indicate that up to 20-30% of patients referred to tertiary clinics with a low-positive antinuclear antibody (ANA) titer (e.g., 1:80 or 1:160) do not suffer from systemic lupus erythematosus (SLE) or any defined connective tissue disease. Misinterpreting these baseline serological variations as definitive evidence of systemic autoimmunity triggers a dangerous cascade of diagnostic momentum. This was precisely the trap in case six, where hand-arm vibration syndrome (HAVS) was mislabeled as evolving systemic sclerosis due to a benign, low-titer ANA decoy.

Misinterpretation of pathognomonic markers and imaging artifacts

True serological phenocopies challenge the absolute specificity of traditional pathognomonic markers. Crystalline silica exposure, as seen in case two, acts as a potent adjuvant that activates the NLRP3 inflammasome, inducing chronic antigenic shedding and a serological profile (anti-Sm and anti-dsDNA) indistinguishable from idiopathic SLE. Similarly, severe systemic inflammation and hypergammaglobulinemia can cause false-positive immunoassay results via non-specific protein binding, a phenomenon that generated the false Anti-HMGCR "myositis" signal in case three.

Furthermore, overreliance on sensitive imaging without biochemical correlation represents a major contemporary pitfall. In case four, chronic fatty infiltration in structural muscular dystrophy created a "pseudoinflammatory" hyperintense signal on T2/STIR sequences, mimicking active myositis edema for three years. Misinterpreting non-specific findings also extends to the "steroid-confirmation fallacy" (case seven), where a dramatic response to low-dose prednisone masked an underlying paraneoplastic polymyalgic syndrome secondary to ovarian carcinoma.

Overdiagnosis and the need for metabolic audits

Finally, our cohort underscores the necessity of rigorous metabolic and nutritional audits before cementing "catch-all" or specialized diagnoses. Literature demonstrates that nearly one-third of patients previously diagnosed with fibromyalgia syndrome (FMS) by primary care physicians do not satisfy the updated American College of Rheumatology (ACR) diagnostic criteria upon expert re-evaluation. multidisciplinary approach, involving rheumatologists, primary care providers, and nutritionists, is essential to address the multifaceted nature of chronic pain and ensure comprehensive care. As shown in case eight, concurrent profound nutritional deficiencies (vitamin D, iron, and B12) can perfectly simulate the chronic pain, fatigue, and cognitive fog of FMS. Similarly, atypical calcium pyrophosphate deposition (CPPD) disease (case one) can act as a metabolic red flag for primary hyperparathyroidism, where borderline hyperuricemia serves as a diagnostic decoy for refractory gout.

Ultimately, these encounters emphasize that a meticulous lifestyle, occupational, and nutritional audit, paired with rigorous clinical reasoning, must remain the primary compass in modern rheumatology. Clinicians must practice active diagnostic vigilance to ensure we treat the actual patient, not just the laboratory or imaging report.

Limitations of the study

This review has several limitations that should be acknowledged. First, it is a retrospective chart review conducted within a single tertiary rheumatology clinical practice, which may introduce selection bias and limit the generalizability of the findings to primary care or non-specialized settings. Second, the small sample size (n = 8) precludes formal epidemiological interpretation regarding the precise prevalence of these specific mimics. Finally, because the analysis relied on existing medical records, longitudinal standardized outcome measures were not uniformly available for all patients. The retrospective nature of this study also introduces the possibility of recall bias and variability in data documentation, which may have affected the completeness of the clinical histories. Despite these limitations, this series serves as a high-yield qualitative framework to enhance clinical reasoning and reduce diagnostic errors.

## Conclusions

Navigating the landscape of modern rheumatology requires a deliberate pause before cementing a diagnosis based solely on classification criteria. While advanced diagnostics offer unprecedented sensitivity, they inadvertently increase the prevalence of diagnostic phenocopies. Ultimately, this series demonstrates that a comprehensive, patient-centered history remains the clinician's primary compass-ensuring we treat the complex biological reality of the individual rather than an isolated laboratory or radiological artifact.

As clinicians, we must remain humble and curious, recognizing that each patient's story is unique and that the correct diagnosis often requires patience, persistence, and a willingness to challenge our own assumptions.
